# Evaluation of Global Genomic DNA Methylation in Human Whole Blood by Capillary Electrophoresis UV Detection

**DOI:** 10.1155/2017/4065892

**Published:** 2017-12-03

**Authors:** Angelo Zinellu, Elisabetta Sotgiu, Stefano Assaretti, Salvatore Sotgia, Panagiotis Paliogiannis, Gianfranco Pintus, Arduino A. Mangoni, Ciriaco Carru

**Affiliations:** ^1^Department of Biomedical Sciences, University of Sassari, Sassari, Italy; ^2^Department of Clinical and Experimental Medicine, University of Sassari, Sassari, Italy; ^3^Department of Biomedical Sciences, College of Health Sciences, Qatar University, Doha 2713, Qatar; ^4^Department of Clinical Pharmacology, College of Medicine and Public Health, Flinders University, Adelaide, SA, Australia

## Abstract

Alterations in global DNA methylation are implicated in various pathophysiological processes. The development of simple and quick, yet robust, methods to assess DNA methylation is required to facilitate its measurement and interpretation in clinical practice. We describe a highly sensitive and reproducible capillary electrophoresis method with UV detection for the separation and detection of cytosine and methylcytosine, after formic acid hydrolysis of DNA extracted from human whole blood. Hydrolysed samples were dried and resuspended with water and directly injected into the capillary without sample derivatization procedures. The use of a run buffer containing 50 mmol/L BIS-TRIS propane (BTP) phosphate buffer at pH 3.25 and 60 mmol/L sodium acetate buffer at pH 3.60 (4 : 1, *v/v*) allowed full analyte identification within 11 min. Precision tests indicated an elevated reproducibility with an interassay CV of 1.98% when starting from 2 μg of the extracted DNA. The method was successfully tested by measuring the DNA methylation degree both in healthy volunteers and in reference calf thymus DNA.

## 1. Introduction

The term “epigenetics” refers to various, potentially heritable, processes that modify gene expression and chromatin structure under exogenous stimuli, with no changes in genetic sequences [1]. Methylation of DNA cytosines of the dinucleotide sequence CpG is one of the major forms of epigenetic modification that plays an important role in gene expression and cellular differentiation [2]. This modified cytosine is designed as the fifth base of human DNA and constitutes approximately 1% of the bases in mammalian genomes [3]. Three active DNA methyltransferases, DNMT1, DNMT3a, and DNMT3b, catalyze the addition of the methyl group from the methyl donor *S*-adenosylmethionine (SAM) to DNA cytosine to form 5-methylcytosine [4, 5]. DNMT1 acts as a “maintenance” methyltransferase by copying the methylation pattern from the parent strand to the daughter strand during DNA replication [4], while DNMT3A/B exhibits de novo activity during cellular transitions [5]. The inclusion of a methylated cytosine in a promoter region has a regulatory effect on gene transcription since it can prevent the binding of transcription factors to DNA [6]. Generally, DNA hypermethylation is associated with gene silencing while hypomethylation is associated with increased transcriptional activity. The available evidence suggests an important role of DNA methylation in common diseases such as cancer, cardiovascular diseases, and neurological disorders [7–9].

Because of the potential impact of both hypo- and hypermethylation of cytosine on health and disease, there is an increasing need for techniques that are able to easily detect and measure DNA methylation. Among the available analytical approaches, acidic hydrolysis of DNA by means of formic acid followed by quantification of the resulting free nucleobases, or enzymatic hydrolysis with nucleotides or nucleosides release, provides significant advantages in terms of speed and simplicity. Once produced, free nucleobases or nucleotides can be measured by a wide variety of methods and techniques such as immunoassays [10], thin-layer chromatography [11], HPLC [12–17], UPLC [18], GC [19–23], or capillary electrophoresis [24–27].

We have previously developed two capillary electrophoresis methods for the quantification of global methylation degree from DNA that was either extracted from whole blood [26] or formalin-fixed and paraffin-embedded [27]. Both methods are characterized by two important features: the first one is their ultrafast analysis (five minutes per run). However, the lower interassay reproducibility (CV = 3.2%) is only achieved when starting from 10 to 20 μg of purified DNA because the narrow uncoated capillary (50 μm id) reduces assay sensitivity. The second one is the significant reduction of DNA consumption, due to the high sensitivity of the field-amplified sample injection (FASI) procedure, but without improving the interday reproducibility (CV = 3.3%). However, it has been reported that, for analytes showing a relatively narrow distribution, the statistical power decreases significantly with increasing measurement imprecision, and that this effect is already evident with a CV interassay of 3% [28]. This implies that a high measurement precision is essential to detect differences between groups or to individuate relationships between studied variables.

Therefore, we describe a new method for the measurement of global DNA methylation, involving formic acid hydrolysis and separation on capillary electrophoresis. The use of a 100 μm uncoated silica capillary significantly increased the method sensitivity, thus reducing the quantity of DNA needed for the analysis. Furthermore, the run buffer used in our experiments guaranteed a high reproducibility with an interassay CV of <2.0% (starting from only 2 μg of the extracted DNA), thus reaching the target value suggested for analytes with a narrow distribution in clinical studies [28].

## 2. Materials and Methods

### 2.1. Chemicals

Cytidine, 5-methylcytidine, BIS-TRIS propane, tris(hydroxymethyl)aminomethane (TRIS), H_3_PO_4_, HCl, formic acid, acetonitrile, and deoxyribonucleic acid from calf thymus were purchased from Sigma-Aldrich, Italia (Milan, Italy). All the nucleosides were prepared as 1 mmol/L stock solutions in Milli-Q gradewater (Millipore, Milford, MA, USA) and stored at −80°C until use.

### 2.2. Participants to the Study and Sample Collection

Eighty-six apparently healthy volunteers (52 males and 34 females) aged between 38 and 86 years (mean 66 ± 13 years), not under any pharmacological therapy or folate, B6, and B12 vitamin supplements, were carefully informed about the nature and purpose of the investigation before giving their voluntary consent to participate. After informed consent was obtained, whole blood was collected by venipuncture in 5 mL EDTA vacutainer tubes and kept frozen at −80°C until use. This study was approved by the ethics committee of Azienda Sanitaria Locale n.1 di Sassari (Italy) (prot. 2175/CE del 21/04/2015).

### 2.3. DNA Extraction and Hydrolysis

Genomic DNA extraction was performed from whole blood using the QIAamp DNA Blood Mini Kit following the instructions of “blood and body fluid protocol” provided by the manufacturer. In particular, the kit uses RNase A, which yields RNA-free DNA. After QIAamp procedure, the extracted DNA was checked for 260 and 260/280 nm UV absorption in order to assess, respectively, DNA concentration and purity. An optical density ratio 260/280 ranging between 1.7 and 1.9 was considered indicative of acceptable purity. The purified DNA was then stored in elution buffer (Buffer AE) at 20°C until further processing in the hydrolysis step.

Two µg of DNA was mixed with formic acid (100 µL final volume and 90% final concentration) and incubated at 130°C for 80 min. After hydrolysis, samples were exsiccated at 60°C under vacuum, and the dry residue containing free bases was dissolved in 100 µL of water for immediate analysis, or stored at −20°C.

### 2.4. Capillary Electrophoresis

Capillary electrophoresis (CE) analysis was performed by a PACE MDQ system equipped with a diode array detector (Beckman Coulter, Milan, Italy), using an uncoated fused-silica capillary with an inner diameter of 100 µm and a total length of 60 cm (50 cm to the detection window), by injecting 270 nL (2 psi × 5 s) of the sample. The UV detector was set at 280 nm, and separation was carried out in a run buffer containing 50 mmol/L BTP phosphate buffer at pH 3.25 and 60 mmol/L sodium acetate buffer at pH 3.60 (4 : 1, *v/v*), 18°C, and 22 kV at normal polarity. After each run, the capillary was rinsed for 1 min with 0.1 mmol/L HCl and equilibrated with run buffer for further 1 min.

### 2.5. Evaluation of DNA Methylation

Cytidine and 5-methylcytidine calibrators were serially diluted four times with water from the top calibrators (cytidine (80 µmol/L) and 5-methylcytidine (4 µmol/L)) to obtain 100 μL of the five calibrators. The latter was dried in a 1.5 mL vial with a screw cap through a speed-vac. Then, 100 µL of 90% formic acid was added, and standards were incubated at 130°C for 80 min. After hydrolysis, samples were exsiccated at 60°C under vacuum, and the dry residue containing standard free bases was dissolved in 100 µL of water for immediate analysis, or stored at −20°C.

Calibration curves were obtained by plotting corrected area (peak area divided by migration time) versus concentration of standard solutions of nucleosides. Linear regression analysis was used to calculate the concentration of Cyt and mCyt in hydrolysed DNA samples. The percentage of methylated to total cytosine (mCyt/tCyt) was calculated using the formula: µmol mCyt/(µmol mCyt + µmol Cyt) × 100.

## 3. Results and Discussion

**Figure 1:**
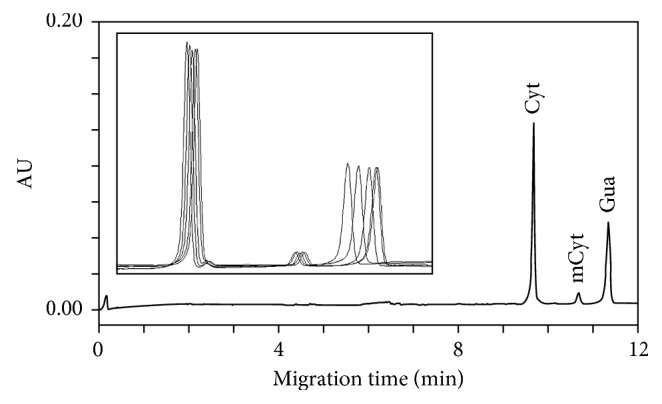
Typical electropherogram of the DNA sample extracted from whole human blood after formic acid hydrolysis obtained with 50 mmol/L BTP phosphate buffer at pH 3.25 as the run buffer. Electrophoretical conditions: uncoated silica capillary, 60 cm × 100 μm id; cartridge temperature, 18°C; voltage, 22 kV; detection, 280 nm; hydrodynamic injection, 270 nL (2.0 psi × 5 s). Panel shows five consecutive runs of hydrolysed DNA. Cyt = cytosine; mCyt = 5-methylcytosine.

Based on our previous experience [26, 27], we performed CE separation of cytosine and methylcytosine by using a run buffer of TRIS phosphate at low pH in a 100 μm id uncoated capillary starting from only 2 μg of DNA. We found that the best compromise between a baseline separation of analytes (cytosine, methylcytosine, and guanosine), shorter analysis times, and acceptable current values was reached by using a 50 mM TRIS phosphate buffer at pH 3.25 and 22 kV and by setting the cartridge temperature at 18°C (Figure 1). However, tests for injection reproducibility failed due to excessive peak shift between runs. Figure 1, describing five consecutive runs of hydrolysed DNA, shows the shift of guanosine toward the mCyt peak. This is also confirmed by the fall of resolution between the two peaks from 5.8 in the first run to 3.1 in the fifth run. The CV value of migration time for five consecutive runs was 0.36% for cytosine, 0.31% for methylcytosine, and 3.1% for guanosine, respectively. Guanosine overlapped the mCyt peak after ten consecutive runs.

In order to improve the assay reproducibility, we used the electrophoretic conditions of our previous CE assay for the measurement of other small basic analytes, in which good separations were reached by using the BTP phosphate buffer as an effective run buffer [29]. However, even if a good separation was reached by using a 50 mmol/L BTP phosphate buffer at pH 3.25, 22 kV, and 18°C, reproducibility was still not acceptable because of an excessive peak shift between runs, similarly to what observed with the TRIS phosphate buffer.

**Figure 2:**
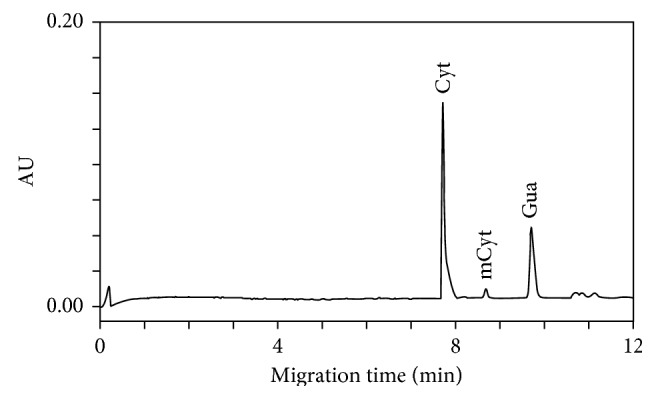
Typical electropherogram of the DNA sample extracted from whole human blood after formic acid hydrolysis obtained with 60 mmol/L sodium acetate buffer at pH 3.60 as the run buffer. Electrophoretical conditions: uncoated silica capillary, 60 cm × 100 μm id; cartridge temperature, 18°C; voltage, 22 kV; detection, 280 nm; hydrodynamic injection, 270 nL (2.0 psi × 5 s). Cyt = cytosine; mCyt = 5-methylcytosine.

We further tested other run buffers such as sodium phosphate, potassium phosphate, sodium acetate, and ammonium acetate using different concentration combinations (between 20 and 100 mmol/L) and pH (between 2.5 and 4.5). We observed good separation and improved reproducibility using 60 mmol/L sodium acetate run buffer at pH 3.60, 22 kV, and 18°C (Figure 2). Although the migration time CV values for five consecutive runs were 0.26% for cytosine, 0.20% for methylcytosine, and 0.30% for guanosine, respectively, the tailing of the cytosine peak indicated the presence of a contaminating element comigrating with Cyt.

**Figure 3:**
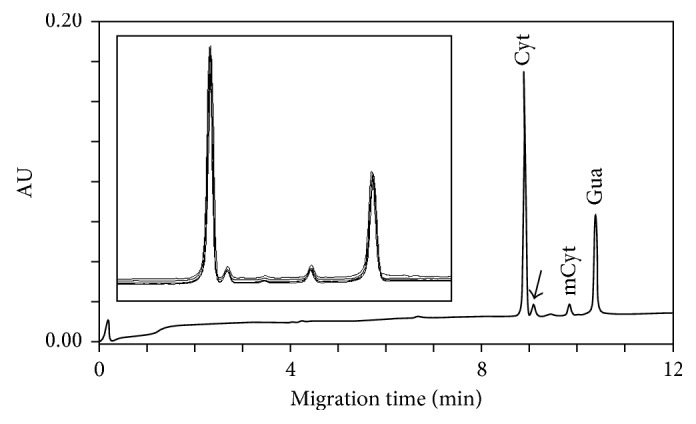
Typical electropherogram of the DNA sample extracted from whole human blood after formic acid hydrolysis obtained with 50 mmol/L BTP phosphate buffer at pH 3.25 and 60 mmol/L sodium acetate buffer at pH 3.60 (4 : 1, *v/v*) as the run buffer. Electrophoretical conditions: uncoated silica capillary, 60 cm × 100 μm id; cartridge temperature, 18°C; voltage, 22 kV; detection, 280 nm; hydrodynamic injection, 270 nL (2.0 psi × 5 s). Arrow indicates the contaminant peak. Panel shows five consecutive runs of hydrolysed DNA. Cyt = cytosine; mCyt = 5-methylcytosine; Gua = guanosine. Peaks were identified by either co-injection with standards and through the analysis of absorbance spectra.

Therefore, we combined the BTP phosphate buffer with the sodium acetate buffer with the aim to obtain the separation performance of the first and the reproducibility of the second. We identified the optimal buffer combination by mixing 50 mmol/L BTP phosphate buffer at pH 3.25 and 60 mmol/L sodium acetate buffer at pH 3.60 (4 : 1, *v/v*), 22 kV, and 18°C. As reported in Figure 3, these conditions allow to separate the cytosine peak from the contaminant peak in the right (identified with a black arrow) and to obtain a good reproducibility, as shown with five consecutive runs of hydrolysed DNA. Migration time CV values for five consecutive runs were 0.30% for cytosine, 0.33% for methylcytosine, and 0.24% for guanosine, respectively. CV values for the corrected peak areas of Cyt and mCyt were about 1%, while the CV for mCyt/tCyt ratio areas was 0.27%.

Similar results, in terms of migration time and selectivity between cytosine and the undefined peak, were observed by mixing 50 mmol/L TRIS buffer at pH 3.25 and 60 mmol/L sodium acetate buffer at pH 3.60 (4 : 1, *v/v*), 22 kV, and 18°C. However, migration time CV values for five consecutive runs were less acceptable at 0.40% for cytosine, 0.51% for methylcytosine, and 1.02% for guanosine, respectively. The CV value for corrected peak areas was 1% for Cyt and 1.59% for mCyt, while the CV for mCyt/tCyt ratio areas was 0.64%.

Due to its superior reproducibility, the combination of 50 mmol/L BTP phosphate buffer at pH 3.25 and 60 mmol/L sodium acetate buffer at pH 3.60 (4 : 1, *v/v*) was selected as the preferred electrolyte run buffer. This buffer composition allowed to obtain a good resolution between all peaks and suitable migration times (less than 11 min) with acceptable current values (115 μA), an electric field of 22 kV, and a capillary cartridge temperature of 18°C.

The calibration curves from six calibration standards of cytosine and 5-methylcytosine, ranging from 2.5 to 80 μmol/L and from 4 to 0.125 μmol/L, respectively, obtained by linear regression analysis, showed a good coefficient of determination (*R*^2^ ≥ 0.99), ensuring a linear response over the concentrations tested. The limits of detection and quantification were derived by analyzing diluted standards of cytosine and 5-methylcytosine. The limit of detection (signal-to-noise ratio (*S/N*) = 3) for cytosine and 5-methylcytosine was 2 nmol/L, whereas the limit of quantification (*S/N* = 10) was 7 nmol/L. The lowest amount of DNA required to evaluate methylation, measured by scalar dilution of the extracted DNA sample, was ∼0.05 μg. The interassay CV value for 5-methylcytosine/total cytosine measured for the DNA extracted from whole blood was 1.98% when starting from 2 μg of DNA, 2.43% when starting from 1 μg of DNA, and 2.91% when starting from 0.5 μg of DNA.  Table 1 presents the interassay CV values of our method compared with other methods reported in literature.

The results obtained from the reference calf thymus DNA analysis, which has a degree of methylation similar to human DNA, were comparable to other assays, with a ratio of 6.48% using our method versus a mean of 6.43 and 6.75% with other methods [12, 16, 17, 21, 22]. This suggests the absence of systematic errors in the measurement process.

**Figure 4:**
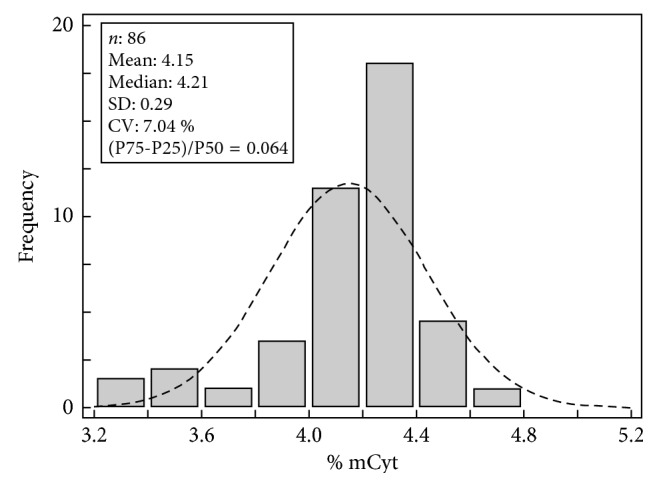
Distribution plots of % mCyt in 86 healthy subjects. The biological variation expressed as interindividual CV was 7.04%, and the ratio between the interquartile difference and the median was 0.064.

Moreover, the DNA methylation degree in 86 healthy volunteers (4.13%) was similar to that reported in literature (between 4.00 and 4.55%) [12, 24]. Similarly to what previously reported [12], the distribution of the mCyt/tCyt ratio values in our study exhibited a moderate negative skewness with values showing a relatively narrow distribution, characterized by a relatively low biological variation (interindividual CV 7%) with a ratio between the interquartile difference and the median of 0.064 (Figure 4). By contrast, in a normal population, both the interindividual CV values (30.3%) and the ratio between the interquartile difference and the median (0.43) are significantly higher for other analytes such as homocysteine [28]. The relatively narrow distribution of the whole blood methylation DNA degree in the general population highlights the importance of developing assay methods that are characterized by high precision. In fact, assays with high CV values may lead to a reduced statistical power in clinical trials and to a significant underestimation of relevant associations in epidemiological studies [28].

## 4. Conclusion

The run buffer selected in our method, containing 50 mmol/L BTP phosphate buffer at pH 3.25 and 60 mmol/L sodium acetate buffer at pH 3.60 (4 : 1, *v/v*), allows a baseline separation of 5-methylcytosine, cytosine, and guanosine after DNA acidic hydrolysis in less than 11 minutes. The BTP phosphate buffer is fundamental to separate the cytosine peak from an undefined contaminant peak, while the sodium acetate buffer guarantees the high reproducibility of both migration time and peak areas. Moreover, the use of a 100 μm id uncoated capillary allows a large-volume injection of the sample, 270 nL versus the 10 nL of our previous CE method (in which a 50 μm id uncoated capillary was used) [26] and a 27-fold increase in sensitivity. The improved sensitivity and the optimized electrophoretic condition ensure a high interassay reproducibility with CV values < 2% (starting from only 2 μg of the extracted DNA), thus significantly improving the interassay reproducibility values previously reported in literature to reach the target value suggested (interassay CV of <3.0%) for analytes with a narrow distribution [28]. The obtained data both from healthy volunteers and from reference calf thymus DNA are in good agreement with previously reported data, demonstrating the absence of systematic errors in the measurement process.

In conclusion, we have described an improved CE-UV detection method for the measurement of global DNA methylation, which showed high sensitivity and superior interday precision versus previously reported methods, making it particularly suitable for routine analysis of large number of samples in clinical studies.

## Figures and Tables

**Table 1:** Interassay precision of the quality control samples calf thymus DNA or human leukocyte (HL) of our CE method compared to other published values.

Interassay CV%	Sample	Analytical method	Reference
3.5	Calf thymus	HPLC-ESI-MS/MS	[12]
5.7	HL DNA	HPLC-ESI-MS/MS	[13]
5.58–6.06	HL DNA	HPLC-MS/MS	[14]
4.92	HL DNA	HPLC-MS/MS	[15]
3.8	Calf thymus	HPLC-UV	[16]
15	Calf thymus	HPLC-UV	[17]
<4	Calf thymus	UPLC-UV	[18]
<15	HL DNA	GC-MS	[19]
7	Calf thymus	GC-MS	[20]
6.5^a^	Calf thymus	GC-MS	[21]
3.0	Calf thymus	GC-FID	[22]
8.2^a^	Calf thymus	GC-ECD	[23]
<5	Calf thymus	CE-LIF	[24]
<5	Calf thymus	CE-UV	[25]
3.2	HL DNA	CE-UV	[26]
3.3	HL DNA	CE-UV	[27]
1.98	HL DNA	CE-UV	Our method

^a^Data published in mol% are converted to 5-methylcytosine/total cytosine ratio.
